# Dietary Lipoic Acid Influences Antioxidant Capability and Oxidative Status of Broilers

**DOI:** 10.3390/ijms12128476

**Published:** 2011-11-29

**Authors:** Peng Chen, Qiu-Gang Ma, Cheng Ji, Jian-Yun Zhang, Li-Hong Zhao, Yong Zhang, Yong-Ze Jie

**Affiliations:** State Key Laboratory of Animal Nutrition, College of Animal Science and Technology, China Agricultural University, Beijing 100193, China; E-Mails: cpneau@yahoo.cn (P.C.); futurezjy@sina.com (J.-Y.Z.); lihongzhao100@126.com (L.-H.Z.); yongzhang208@163.com (Y.Z.); jieyongze443@gmail.com (Y.-Z.J.)

**Keywords:** lipoic acid, broiler, antioxidant capability

## Abstract

The effects of lipoic acid (LA) on the antioxidant status of broilers were investigated. Birds (1 day old) were randomly assigned to four groups and fed corn-soybean diets supplemented with 0, 100, 200, 300 mg/kg LA, respectively. The feeding program included a starter diet from 1 to 21 days of age and a grower diet from 22 to 42 days of age. Serum, liver and muscle samples were collected at 42 days of age. For antioxidant enzymes, superoxide dismutase (SOD) and glutathione peroxidase (GSH-Px) activity in serum, liver and breast muscle significantly increased in chickens fed with LA. The concentration of malondiadehyde (MDA), an indicator of lipid peroxidation, was significantly lower in serum, liver and leg muscle in birds that received LA than in the control group. Treatments with LA significantly increased glutathione (GSH) content in liver and increased α-tocopherol content in leg muscle as compared to the control. These results indicate that dietary supplementation with 300 mg/kg LA may enhance antioxidant capability and depress oxidative stress in broilers.

## 1. Introduction

α-Lipoic acid (LA), also known as thioctic acid (chemical name: 1,2-dithiolane-3-valeric acid or 6,8-dithio-octanoic acid) is a naturally occurring compound widely distributed among microorganisms, plants and animals [1]. Lipoic acid complex with lysine, called lipoamide, functions as an essential co-factor in the mitochondrial dehydrogenase complexes that catalyse the oxidative decarboxylation of α-keto acids [2,3]. In the course of this reaction, LA is reduced to dihydrolipoic acid (DHLA) and the two substances operate as a redox couple. Due to their roles as biological thiol antioxidants, LA and its reduced form, DHLA, have gained considerable attention. Several features which make them outstanding antioxidants have been well documented in various *in vitro* and *in vivo* systems [1,4]. First, LA and DHLA act as a redox couple which can regenerate other natural antioxidants such as glutathione (GSH), vitamin C, coenzyme Q10, ubiquinone and vitamin E to protect the integrity of cell membranes [5]. Second, because LA and DHLA are amphipathic molecules and able to scavenge free radicals both in hydrophilic and lipophilic environments, their biological functions are not limited solely to one environment [6]. In addition to reactive oxidant species (ROS) scavenging, both LA and DHLA are able to chelate a wide variety of metals that are associated with increased production of free radicals [7].

So over the last two decades, the use of LA as antioxidant in food supplements to prevent or cure human diseases associated with oxidative stress has become immensely popular [8,9]. Our laboratory’s previous study showed that LA at 300, 600 or 900 mg/kg concentrations can enhance antioxidative ability of broilers; however at the expense of growth performance [10] due to the fact that LA can reduce food intake and body weight [11] especially at high supplementation amounts. Thus, this study tested whether supplementation of LA in lower levels can also improve antioxidant property.

## 2. Materials and Methods

### 2.1. Birds and Experimental Design

A total of 192 1-day-old Ross male broiler chicks (*Gallus domesticus*) were obtained from a commercial hatchery (Chia Tai Co., Ltd, Hebei, China) and randomly allocated to 4 treatments, each with 4 replicates consisting of 12 chicks. The four dietary treatments were basal corn-soybean meal diet (mash form) supplemented with 0 (control), 100, 200, 300 mg/kg dl-α-lipoic acid (Sigma Chemical, St. Louis, MO, USA) which was mixed in the feed, respectively. The chicks were reared under commercial management practice, and fed a commercial two-phase broiler diet and provided with water ad libitum throughout the rearing period. Starter and grower diets were offered to the birds from 0 to 3 weeks of age and from 4 to 6 weeks of age, respectively. Ingredients and nutrient composition of the basal diet was designed according to the commercial diets popularly applied by broiler farmers in China. All essential nutrients in the basal diet (Table 1) met or slightly lowered than nutrient requirements of National Research Council [12]. The animal care protocol in this experiment was approved by the Animal Welfare Committee of China Agricultural University. At the end of the experiment, twelve broilers per treatment group (three birds per replicate) were bled via brachial vein for serum sample and then slaughtered and dissected by a trained team. Blood was centrifuged at 1400 g at 8 °C for 30 min and serum was separated. Serum, liver, breast (pectoralis major) and leg muscle (thigh muscle) were collected and snap-frozen in liquid nitrogen. Frozen tissues were stored at −70 °C prior to analysis.

### 2.2. Tissue Preparation

Immediately upon euthanization, the liver and muscles were excised and placed in 5 mL cryotubes, then divided into two sub-sample groups, frozen (liquid N_2_), and then stored at 80 °C. Half of the tubes were used for α-tocopherol assays and the others were kept for further analyses of glutathione (GSH), and superoxide dismutase (SOD), total antioxidant capability (T-AOC), glutathione peroxidase (GSH-Px) and the content of malondialdehyde (MDA). Tissue samples were thawed and homogenized on ice-cold saline buffer (10 mM; pH = 7.4) (1:10, wt/v) with an Ultra-Turrax (T8, IKA-Labortechnik, Staufen, Germany) to form homogenates at the concentration of 0.1 g/mL for further analysis.

### 2.3. Assay of Antioxidant Indices in Serum, Liver and Muscle

The homogenate was centrifuged at 1000 g for 10 min at 4 °C. Then, the supernatant and serum already prepared were subjected to the measurement of SOD, T-AOC, GSH-Px, GSH and MDA levels using colorimetric methods with a spectrophotometer (Biomate 5, Thermo Electron Corporation, Rochester, NY, USA). The assays were conducted with the assay kits obtained from Nanjing Jiancheng Insititute of Bioengineering (Nanjing, Jiangsu, China) and the procedures accordingly. Briefly, Activity of SOD was measured by the xanthine oxidase method, which monitors the inhibition of reduction of nitro blue tetrazolium by the sample [13]. The T-AOC was measured by the method of ferric reducing-antioxidant power assay [14] and detected at 520 nm with the spectrophotometer. Activity of GSH-Px was detected with 5,5′-dithiobis-*p*-nitrobenzoic acid, and the change of absorbance at 412 nm was monitored using a spectrophotometer [15]. GSH was estimated on the basis of the transformation of thioesters with GSH into a chromophoric thione measured at 400 nm. The MDA level was analyzed with 2-thiobarbituric acid, monitoring the change of absorbance at 532 nm with the spectrophotometer [16]. Enzyme activity was expressed as units per milligram of protein for tissues and units per milliliter for serum. Protein content of supernatants was determined using the Coommasie Brilliant Blue G250 (Sigma Chemical, St. Louis, MO, USA) assay with bovine serum albumin.

### 2.4. α-Tocopherol Content

Tissue samples were first ground individually in a preparation containing 700 mL NaCl (5% w/vol), 1 mL ethanol and 2.3 mL hexane, then centrifuged at 9000 rpm for 6 min. The supernatant was extracted from the mixture with hexane, and then pooled and evaporated (N_2_ atmosphere) on a heated (65 °C) dry-block. The dry matter was dissolved in a mixture of dichloromethane and methanol and centrifuged for 3 min. Extracts were injected into a HPLC system (Shimadzu Liquid Chromatograph, LC-10AD, Japan Spectroscopic Co. Ltd.). The mobile phase used here was a solution-containing methanol and water (97:3 v/v) adjusted to a flow rate of 1.1 mL/min. Fluorescence detection of α-tocopherol involved excitation and emission wavelengths of 295 and 330 nm, respectively. Standard solutions of α-tocopherol in methanol were used for instrument calibration.

### 2.5. Statistical Analysis

All data are expressed as means and standard deviations among means (*P* < 0.05) and were determined by SPSS [17]. Comparison between groups was by one-way ANOVA. The linear and quadratic effect of LA among treatments was analyzed using a contrast statement. Where appropriate, Duncan’s multiple range test was used to determine the significance of differences between treatment means. Values of *P* < 0.05 were considered significant.

## 3. Results

Table 2 shows there is no difference (*P* > 0.05) among groups in average daily feed intake (ADFI). The average daily LA intake (ADLI) and the average daily LA intake on the basis of metabolic body weight (ADLI/BW^0.75^) in broiler chickens is also shown in this table.

Table 3 presents serum antioxidant enzyme activities and lipid peroxidation levels. Treatment of birds with 200 and 300 mg/kg of LA caused an increase of GSH-Px activity by 19.0% (*P* < 0.05) and 23.2% (*P* < 0.05). The activity of SOD was greatly (*P* < 0.05) elevated in the 300 mg/kg of LA treatment compared with the control. The activity of SOD and GSH-Px increased linearly (*P* < 0.001) as LA increased. Activities of T-AOC were not affected (*P* > 0.05) by dietary LA. The levels of MDA were significantly (*P* < 0.05) decreased in 200 and 300 mg/kg of LA treatments compared with the control and decreased linearly (*P* < 0.001) as LA increased.

Table 4 shows activities of antioxidant enzymes and lipid peroxidation levels in liver. Treatments with LA linearly (*P* < 0.001) increased (17.6%, 27.9% and 31.6%, respectively) hepatic SOD activity as compared to the control. Chicks fed 300 mg/kg of LA diet had more GSH-Px activities than chicks fed on the control diet (*P* < 0.05). Also, MDA values in liver of chicks fed on 100, 200 and 300 mg/kg of LA diet were significantly decreased by 43.2%, 44.1% and 45.7% compared with chicks fed on the control diet (*P* < 0.05) and also in a linear increase manner (*P* = 0.008). Activities of T-AOC were not affected (*P* > 0.05) by dietary LA.

Tables 5 and 6 show activities of antioxidant enzymes and lipid peroxidation levels in breast and leg muscles. Dietary 200 or 300 mg/kg of LA significantly (*P* < 0.05) increased SOD activity compared with the control or 100 mg/kg of LA treatment; however, in breast muscle there is no differences observed among groups. Both in leg and breast muscle, SOD activity increased linearly as LA increased. Although T-AOC activity increased linearly (*P* < 0.05), it was not affected by LA supplement. Supplementation with LA significantly (*P* < 0.05) increased (22.5%, 25.5% and 29.4%, respectively) GSH-Px activity in breast muscle compared with the control, and significant linear responses were detected. The levels of MDA in leg muscle were significantly (*P* < 0.05) decreased in 200 and 300 mg/kg of LA treatments compared with control; however, LA did not affect MDA level in breast muscle.

Table 7 shows levels of GSH in liver and muscles of broilers. In liver, treatments with LA significantly increased (23.1%, 30.6% and 36.1%, respectively) GSH content as compared to the control. This response was also linear (*P* < 0.001). LA did not affect GSH content either in breast or leg muscle.

Table 8 shows levels of α-tocopherol in muscles of broilers. α-tocopherol content in leg muscle from birds supplemented with LA increased (*P* < 0.05) in a linear manner (*P* < 0.001) compared with the control, however, there was no significant difference among the groups with regard to α-tocopherol concentration in breast muscle.

## 4. Discussion

In living animals, oxidative stress constitutes an important mechanism that causes several pathologies that affect poultry growth [18]. In food for humans, oxidative rancidity represents one of the major causes of deterioration that is responsible for loss in appearance and nutritional value. Although the clinical manifestation of chronic pathologies related to oxidative stress in poultry is limited by their life-span (generally defined by their growth for productive purposes), the benefits of supplementing animal diets for human consumption (*i.e.*, poultry, pigs) with antioxidants are far more than only to secure food preservation [19]. In recent years an increasing number of studies have pointed out the antioxidant properties benefits of LA [8], however unlike in humans or experimental animals, the effect of LA on biomarkers of oxidative stress and antioxidant status has not yet been systematically studied in birds. Therefore, to explore the effect of dietary LA on antioxidant capability and oxidative status of broilers, the specific activity of antioxidant enzymes, non-enzymatic antioxidants and the level of lipid peroxidation in the serum and liver as well as muscle were examined in the current study.

The chosen supplemental amount of LA in this study is similar to human usual doses of LA (300–600 mg per day; body weight, 75 kg), if scaled on the level of metabolic body size (BW_kg_^0.75^). In addition, no significant difference among groups in ADFI was observed (Table 2). Moreover, the average daily LA intake (ADLI) and average daily LA intake on the basis of metabolic body weight (ADLI/BW^0.75^) were linearly increased. These results indicate that we can exclude the possibility that feed intake is depressed by LA, decreasing the LA intake, and then influence the effects of LA on antioxidant capability of birds.

Exogenously supplied LA, a non-protein thiol, is readily taken up by a variety of cells and tissues in which it is rapidly reduced by NADH- or NADPH-dependent enzymes to DHLA [1]. Lipoic acid is unusual among endogenous and exogenous antioxidants as it is a potent direct reactive species quencher in its oxidized form. Lipoic acid is capable of scavenging hydroxy radicals, hypochlorous acid and singlet oxygen, but not superoxide or peroxyl radicals [1]. DHLA is a strong reductant with a standard reduction potential of 20.32 V [20]. It can be stated that the dithiol, apart from effectively clearing the hydroxyl, peroxyl and superoxide radicals [21], also enhances the levels of these antioxidants GSH, vitamins C and E by providing the reducing milieu and regenerating them via the reduction of their radicals [22].

There is growing evidence that LA may act indirectly to maintain cellular antioxidant status by enhancing the synthesis of non-enzymatic antioxidants such as reduced glutathione, vitamin C and α-tocopherol (vitamin E) [8]. For instance, reports show that LA increases intracellular α-tocopherol levels. Aged rats exhibit a decline in α-tocopherol concentrations, but dietary LA restored α-tocopherol levels and lowered the rate of oxidant production to the level seen in young rats [23]. In current study inclusion of LA into the diet caused a significant dose-dependent response in terms of leg muscle α-tocopherol level. This may be due to the suppression of α-tocopherol consumption probably through the enhanced ROS scavenging by LA as well as due to recycling α-tocopherol by regenerating ubiquinol, vitamin C, and GSH during the redox cycles of LA and DHLA [24]. This explanation is in agreement with a study of Arivazhagan *et al.* [25], who reported that the administration of LA to aged rats increased the concentrations of GSH and vitamins C and vitamin E. In contrast to the high α-tocopherol level in leg muscle, the α-tocopherol concentration was unchanged in the breast muscle. On one hand, possibly because breast muscle has a relatively lower capillary density and blood flow rate than leg muscle. On the other hand, breast muscle contains much less lipid than thigh muscle, so maybe α-tocopherol concentration in breast muscle was not sensitive enough to pick up small differences.

Vitamin E functions biologically as a membrane-specific scavenger of free radicals, and GSH-Px functions as a destroyer of peroxides [26]. Yang *et al*. [27] reported that both excess and deficient amounts of vitamin E significantly depress the activity of GSH-Px in liver and plasma of rats. Lin *et al*. [28] also reported that plasma GSH-Px activities of pullets were depressed by higher levels of supplemental vitamin E, suggesting the activities of GSH-Px and vitamin E have complementary roles against oxidative agents. In our study, GSH-Px activity in leg muscle and α-tocopherol concentration in breast muscle were both decreased (*P* > 0.05) by LA. We speculate the high leg muscle α-tocopherol concentration might have reduced the susceptibility to lipid peroxidation (*P* < 0.05) and thus lowered GSH-Px activity, and also the findings that no difference observed in MDA level and high GSH-Px activity in breast muscle may be attributed to the unremarkable α-tocopherol concentration.

Glutathione serves as a sensitive marker of oxidative stress and it plays an important role in maintaining the integrity of the cell system. GSH is involved in several reactions in the body and is one of the most prominent non-enzymatic antioxidants [29]. Recent studies have uncovered the mechanism by which LA increases cellular GSH [30,31]. DHLA markedly improves cysteine availability within the cell, resulting in accelerated GSH synthesis [32]. In view of this mechanism of action of LA, it may be expected that the effect of LA on tissue GSH would be most marked in organs that have a high activity of GSH-synthesizing enzymes. We observed that LA supplementation increased the GSH level of liver. Such observations may be explained by the fact that liver have remarkably high activity of GSH-synthesizing enzymes [33,34].

In our study, increased SOD and GSH-Px activity in serum, liver and leg muscle in LA-fed chicks were observed. Similar findings were reported by Zhang *et al*. [10] and Srilatha *et al*. [35] in broilers and by Seaton *et al*. [36] in rats. The enzymatic antioxidant defence systems are the natural protectors against lipid peroxidation. They include superoxide dismutase and glutathione peroxidase [37]. First, SOD converts the superoxide anion to hydrogen peroxide in a cellular antioxidant reaction. Thereafter, GSH-Px detoxify hydrogen peroxide produced [38]. It has been suggested that LA can reduce oxidized GSH and increase the GSH status, which in turn exhibits increased free radical scavenging property, so LA indirectly influences the activity of SOD thereby preventing the deleterious effect of superoxide radical formed. Apart from this, the increased intracellular GSH content might also activate the GSH dependant enzyme GSH-Px thereby preventing the accumulation of H_2_O_2_. When GSH-Px succeeded eliminating H_2_O_2_ from the cell, the fallen H_2_O_2_ has been shown to cause activation of SOD [39]. The increase in the activities of these enzymes could reflect the positive effects of LA on this finely balanced antioxidant system. From these observations, we suggested that dietary LA enhances the capacity of scavenging free radicals.

Elevation in the activities of these antioxidant enzymes was discernible after LA supplementation in birds and may, therefore, account for the observed reduced level of MDA in serum and liver. MDA is formed as an end product of lipid peroxidation and therefore the extent of lipid peroxidation by ROS can be monitored by MDA levels [40]. Hence, the reduced serum MDA level in LA-supplemented as compared with control broilers indicated that lipid peroxidation was reduced by LA via enhancing antioxidative action. The effect of LA on MDA levels found in our study confirms previously reported findings of other investigators [41–43].

Lipid peroxidation is the major cause for impaired meat quality through production of off-flavors and odors, destruction of polyunsaturated fatty acids, fat-soluble vitamins and pigments, and the production of toxic compounds such as peroxides and aldehydes [44]. Antioxidants are commonly added to broiler diets to improve antioxidant status and prevent lipid oxidation in the meat. Savitha *et al.* [45] reported that the supplementation of LA with other antioxidants decreased the levels of thiobarbituric acid reactive substances and improved the antioxidant status in skeletal muscles. In our study, dietary LA supplementation did not change the MDA value in breast muscle, but at 200 or 300 mg/kg concentration, lowered the MDA value in leg muscle. In addition, we found that MDA values in leg muscle from all dietary groups were always higher than those in breast muscle from the equivalent dietary treatments, reinforcing the fact that leg muscle tissues were more susceptible to oxidation than breast muscle tissues [46]. A possible explanation is that the leg muscle mainly comprised of slow twitch fibers has an increased oxygen consumption rate and higher level of ROS generation in comparison with the breast muscle mainly comprised of fast twitch fiber fibers. Furthermore, the significant increase in α-tocopherol concentration in leg muscle may have also contributed to the decreased MDA level in this study.

## 5. Conclusions

Most studies with LA until now were carried out in animals other than in livestock, and in organs and systems other than the skeletal muscle which is particularly susceptible to peroxidation due to the simultaneous presence of high levels of polyunsaturated fatty acids. In the present work, we demonstrated that LA supplementation improved antioxidative status of broilers by elevating activity of antioxidant enzymes and reducing peroxidation products, and LA supplementation inhibited lipid peroxidation in skeletal muscles by increasing both the α-tocopherol level and SOD and GSH-Px activities in broilers. This effect was especially profound in the leg muscle, which is particularly sensitive to oxidative stress.

## Figures and Tables

**Table 1:** Ingredients and nutrient composition of the basal diet.

Items	Starter Phase	Grower Phase
Ingredients (%)		
Corn	46.90	55.50
Corn gluten meal	5.70	2.40
Extruded soybean	20.00	16.00
Soybean meal	20.00	19.00
Limestone	1.20	1.30
Dicalcium phosphate	1.70	1.20
Sodium chloride	0.30	0.30
Corn oil	2.70	2.80
Premix *	1.00	1.00
Bentonite	0.50	0.50
Nutrient composition (Calcaulated)		
ME (MJ/kg)	13.24	13.24
CP (%)	22.7	19.5
Ca (%)	0.98	0.88
Total phosphorus (%)	0.64	0.54
Available phosphorus (%)	0.44	0.35
Met (%)	0.50	0.38
Met + Cystine (%)	0.90	0.73
Lys (%)	1.12	1.00

* The vitamin and mineral premix supplied the following per kilogram of diet: vitamin A, 15,000 IU; cholecalciferol, 3000 IU; vitamin E, 20 IU; vitamin K_3_, 2.18 mg; thiamine, 2.15 mg; riboflavin, 8.00 mg; pyridoxine, 4.40 mg; vitamin B_12_ 0.02 mg; Calcium pantothenate, 25.60 mg; nicotinic acid, 65.80 mg; folic acid, 0.96 mg; biotin, 0.20 mg; Fe, 109.58 mg; Cu, 8.14 mg; Zn, 78.04 mg; Mn, 105.00 mg; I, 0.34 mg; Se, 0.14 mg; choline chloride, 1500 mg.

**Table 2:** Feed intake on the basis of metabolic body weight in broiler chickens subjected to dietary supplementation of lipoic acid.

Item 1	Lipoic Acid, mg/kg				SEM	Contrasts 2	
	
	0	100	200	300		Linear	Quadratic
ADFI, days 1–21, g·day^−1^	45.70	45.50	47.90	45.10	0.13	0.864	0.458
ADFI, days 22–42, g·day^−1^	143.30	145.60	144.80	144.5	0.13	0.510	0.303
ADLI, days 1–21, mg·day^−1^	0.00	4.55	9.57	13.54	0.20	<0.001	<0.001
ADLI, days 22–42, mg·day^−1^	0.00	14.56	28.97	43.36	0.27	<0.001	<0.001
ADLI/BW^0.75^, days 1–21, mg·day^−1^·kg^−1^	0.00	10.27	20.99	30.56	0.47	<0.001	<0.001
ADLI/BW^0.75^, days 22–42, mg·day^−1^·kg^−1^	0.00	11.62	23.08	34.67	0.26	<0.001	<0.001

1 ADFI = average daily feed intake; ADLI = average daily lipoic acid intake; BW = body weight;

2 Dietary lipoic acid level effects were tested using linear and quadratic contrasts.

**Table 3:** Activities of antioxidant enzymes and lipid peroxidation levels in broiler serum at 42 days of age.

Item 1	Lipoic Acid, mg/kg				SEM	Contrasts 2	
	
	0	100	200	300		Linear	Quadratic
SOD (U/mL)	153.25 b	172.29 a,b	178.81 a,b	183.62 a	1.85	<0.001	0.181
T-AOC (U/mL)	10.22	10.25	10.43	10.61	0.45	0.083	0.433
GSH-Px (U/mL)	205.33 b	225.54 a,b	244.27 a	252.87 a	4.32	<0.001	0.264
MDA (nmol/mL)	4.14 b	3.94 b	3.26 a	3.11 a	0.13	<0.001	0.215

a,b Means within a ROW with no common superscript differ significantly (*P* < 0.05);

1 SOD = superoxide dismutase; T-AOC = total antioxidant capability; GSH-Px = glutathione peroxidase; MDA = malondiadehyde;

2 Dietary lipoic acid level effects were tested using linear and quadratic contrasts.

**Table 4:** Activities of antioxidant enzymes and lipid peroxidation levels in broiler liver at 42 days of age.

Item 1	Lipoic Acid, mg/kg				SEM	Contrasts 2	
	
	0	100	200	300		Linear	Quadratic
SOD (U/mg pro)	221.36 b	260.21 a	283.14 a	291.40 a	3.12	<0.001	0.403
T-AOC (U/mg pro)	5.42	5.56	5.48	5.69	0.23	0.323	0.153
GSH-Px(U/mg pro)	5.72 b	6.46 a,b	6.19 a,b	7.46 a	0.13	0.635	0.416
MDA(nmol/mg pro)	3.24 b	1.84 a	1.81 a	1.76 a	0.06	0.008	0.976

a,b Means within a row with no common superscript differ significantly (*P* < 0.05);

1 SOD = superoxide dismutase; T-AOC = total antioxidant capability; GSH-Px = glutathione peroxidase; MDA = malondiadehyde;

2 Dietary lipoic acid level effects were tested using linear and quadratic contrasts.

**Table 5:** Activities of antioxidant enzymes and lipid peroxidation levels in broiler leg muscle at 42 days of age.

Item 1	Lipoic Acid, mg/kg				SEM	Contrasts 2	
	
	0	100	200	300		Linear	Quadratic
SOD (U/mg pro)	125.30 b	130.47 b	152.24 a	159.52 a	1.47	<0.001	0.384
T-AOC (U/mg pro)	1.32	1.41	1.48	1.64	0.05	0.035	0.423
GSH-Px (U/mg pro)	1.75	1.73	1.70	1.68	0.05	0.032	0.649
MDA (nmol/mg pro)	2.38 b	2.25 a,b	1.89 a	1.86 a	0.08	<0.001	0.256

a,b Means within a row with no common superscript differ significantly (*P* < 0.05);

1 SOD = superoxide dismutase; T-AOC = total antioxidant capability; GSH-Px = glutathione peroxidase; MDA = malondiadehyde;

2 Dietary lipoic acid level effects were tested using linear and quadratic contrasts.

**Table 6:** Activities of antioxidant enzymes and lipid peroxidation levels in broiler breast muscle at 42 days of age.

Item 1	Lipoic Acid, mg/kg				SEM	Contrasts 2	
	
	0	100	200	300		Linear	Quadratic
SOD (U/mg pro)	79.45	84.65	88.31	92.12	1.12	0.004	0.774
T-AOC (U/mg pro)	0.88	0.89	0.98	0.94	0.03	0.023	0.547
GSH-Px (U/mg pro)	1.02 b	1.25 a	1.28 a	1.32 a	0.04	<0.001	0.642
MDA (nmol/mg pro)	2.69	2.55	2.51	2.52	0.09	0.031	0.605

a,b Means within a row with no common superscript differ significantly (*P* < 0.05);

1 SOD = superoxide dismutase; T-AOC = total antioxidant capability; GSH-Px = glutathione peroxidase; MDA = malondiadehyde;

2 Dietary lipoic acid level effects were tested using linear and quadratic contrasts.

**Table 7:** Levels of glutathione (μmol/g of tissue) in liver and muscles of broilers at 42 days of age.

Tissue	Lipoic Acid, mg/kg				SEM	Contrasts 1	
	
	0	100	200	300		Linear	Quadratic
Liver	4.77 b	5.87 a	6.23 a	6.49 a	0.18	<0.001	0.194
Breast muscle	0.93	0.98	0.98	1.04	0.04	0.104	0.364
Leg muscle	1.31	1.29	1.42	1.54	0.06	0.235	0.573

a,b Means within a row with no common superscript differ significantly (*P* < 0.05);

1 Dietary lipoic acid level effects were tested using linear and quadratic contrasts.

**Table 8:** Levels of α-tocopherol (μg/g of tissue) in muscles of broilers at 42 days of age.

Tissue	Lipoic Acid, mg/kg				SEM	Contrasts 1	
	
	0	100	200	300		Linear	Quadratic
Breast muscle	0.75	0.74	0.71	0.72	0.02	0.193	0.598
Leg muscle	2.01 b	5.95 a	6.04 a	6.12 a	0.20	<0.001	0.437

a,b Means within a row with no common superscript differ significantly (*P* < 0.05);

1 Dietary lipoic acid level effects were tested using linear and quadratic contrasts.
